# Superior bioavailability of the calcium salt form of β-hydroxy-β-methylbutyrate compared with the free acid form

**DOI:** 10.1007/s00726-023-03369-z

**Published:** 2024-04-02

**Authors:** Heitor Rodrigues Ribeiro, Felipe Gregório Jardim, Miriam Sanz Roldán, Vitor de Salles Painelli, Vinicius da Eira Silva, Aline Cristina Capparelli Tritto, Andressa Formalioni, Giovani Boldrini Custoias, Wagner Ribeiro Pereira, Marina Yazigi Solis, Felipe Carvalho, Ernani Pinto Junior, Guilherme Giannini Artioli

**Affiliations:** 1 Applied Physiology & Nutrition Research Group, University of São Paulo, São Paulo, Brazil; 2 Rheumatology Division, Faculdade de Medicina, Hospital das Clínicas HCFMUSP, Universidade de São Paulo, São Paulo, Brazil; 3 Faculdade de Ciências Farmacêuticas, Universidade de São Paulo (USP), São Paulo, Brazil; 4 Strength Training Study and Research Group, Institute of Health Sciences, Paulista University UNIP, São Paulo, Brazil; 5 Department of Biomedical Physiology and Kinesiology, Simon Fraser University, Burnaby, Canada; 6 Centre for Bioscience, Department of Life Sciences, Manchester Metropolitan University, 215 John Dalton Building, Chester Street, Manchester, Lancashire M1 5DG UK

**Keywords:** Beta-hydroxyisovaleric acid, Absorption, Pharmacokinetics, Performance-enhancing substances

## Abstract

We investigated the bioavailability of the calcium salt (HMB-Ca) and the free acid (HMB-FA) forms of β-hydroxy-β-methylbutyrate (HMB). Sixteen young individuals received the following treatments on three different occasions in a counterbalanced crossover fashion: (1) HMB-FA in clear capsules; (2) HMB-Ca in gelatine capsules; (3) HMB-Ca dissolved in water. All treatments provided 1 g of HMB. Blood samples were taken before and on multiple time points following ingestion. The following parameters were calculated: peak plasma (Cmax), time to peak (Tmax), slope of HMB appearance in blood, area under the curve (AUC), half-life time (*t*_*1/2*_) and relative bioavailability (HMB-Ca in water set as reference). All treatments led to rapid and large increases in plasma HMB. HMB-Ca in capsules and in water showed similar plasma HMB values across time (*p* = 0.438). HMB-FA resulted in lower concentrations vs*.* the other treatments (both *p* < 0.001). AUC (HMB-Ca in capsules: 50,078 ± 10,507; HMB-Ca in water: 47,871 ± 10,783; HMB-FA: 29,130 ± 12,946 µmol L^−1^ × 720 min), Cmax (HMB-Ca in capsules: 229.2 ± 65.9; HMB-Ca in water: 249.7 ± 49.7; HMB-FA: 139.1 ± 67.2 µmol L^−1^) and relative bioavailability (HMB-Ca in capsules: 104.8 ± 14.9%; HMB-FA: 61.5 ± 17.0%) were lower in HMB-FA vs. HMB-Ca (all *p* < 0.001). HMB-Ca in water resulted in the fastest Tmax (43 ± 22 min) compared to HMB-Ca in capsules (79 ± 40 min) and HMB-FA (78 ± 21 min) (all *p* < 0.05), while *t*_*1/2*_ was similar between treatments. To conclude, HMB-Ca exhibited superior bioavailability compared to HMB-FA, with HMB-Ca in water showing faster absorption. Elimination kinetics were similar across all forms, suggesting that the pharmaceutical form of HMB affects the absorption rates, but not its distribution or elimination.

## Introduction

Beta-hydroxy-β-methylbutyrate (HMB) is an organic acid derived from the essential amino acid leucine. The endogenous synthesis of HMB is rate limited (Koevering and Nissen 1992), involving the conversion of leucine to α-ketoisocaproate, primarily in the skeletal muscle (Holeček 2017), in a reaction catalysed by the enzyme BCAA aminotransferase. The resulting α-ketoisocaproate is then converted to HMB in the liver by the enzyme ketoisocaproate dioxygenase (Sabourin and Bieber 1983). Only ~ 2–10% of the leucine metabolism is directed towards HMB formation (van Koevering et al. 1992); therefore, fasting plasma HMB concentration is typically low in humans, in the low micromolar range (Wilkinson et al. 2013). The ingestion of supplemental doses of leucine increases plasma HMB, but to a far lower extent than that elicited by HMB ingestion (Wilkinson et al. 2013), leading to the assumption that HMB supplementation is the most effective way to induce substantive plasma elevations in HMB.

HMB has long been of interest to athletic as well as clinical populations due its anabolic and anti-catabolic properties. HMB supplementation has been shown to increase muscle protein synthesis, which seems to result from a direct stimulation of mammalian target of the rapamycin pathway (Wilkinson et al. 2013, 2018). Evidence also suggests that HMB can attenuate exercise-induced muscle damage (Knitter et al. 2000), potentially leading to improved muscle recovery following exercise, although this has been disputed (Tritto et al. 2019). Moreover, studies in animals (Kovarik et al. 2010; Baptista et al. 2013) and in humans (Wilkinson et al. 2013) have shown that HMB supplementation can suppress muscle protein breakdown, thereby exerting anti-atrophic effects in conditions of exacerbated skeletal muscle catabolism (Prado et al. 2022; Mirza et al. 2014). Human studies showed that HMB can counteract age-related muscle loss (Oktaviana et al. 2019) and muscle wasting in conditions such as bed rest (Deutz et al. 2013) and cancer (Clark et al. 2000). The potentially beneficial effects of HMB supplementation on muscle strength and muscle mass in conditions of exacerbated muscle loss has been supported by meta-analyses (Wu et al. 2015; Bear et al. 2019), although controversy still exists as to whether HMB can effectively support muscle growth in healthy individuals undergoing resistance training, with current evidence not supporting its use for enhancing training adaptations in young, healthy, trained individuals (Tritto et al. 2019; Jakubowski et al. 2020; Phillips et al. 2022).

The controversial findings in the HMB literature have been in part attributed to the pharmaceutical form of HMB. Two forms of HMB are available for human use, namely the calcium salt (HMB-Ca) and the free acid (HMB-FA) forms. HMB-FA is assumed to have superior effects on muscle anabolism compared with HMB-Ca due to its improved bioavailability (Wilkinson et al. 2013; Wilson et al. 2013a, b, 2014). However, only three studies have directly compared the pharmacokinetic profile of HMB-FA with HMB-Ca, two of them being conducted in humans (Fuller et al. 2011, 2015) and one in rats (Shreeram et al. 2014). The two human studies (Fuller et al. 2011, 2015) showed faster increases in plasma HMB, higher peak concentrations and area under the curve following HMB-FA ingestion compared to HMB-Ca. In contrast, the study with rats showed superior bioavailability of HMB-Ca over HMB-FA across three different doses. A more recent study in humans also indicated excellent bioavailability of orally ingested HMB-Ca (Wilkinson et al. 2018). To address these controversies and further the understanding of the pharmacokinetic properties of both forms of HMB, we sought to investigate whether the pharmaceutical form of HMB influences its bioavailability and pharmacokinetic profile in humans.

## Methods

### Study design

In this crossover pharmacokinetic study, male and female participants visited the laboratory on three different occasions to receive one of the following treatments: (1) 1 g of HMB-FA, or (2) HMB-Ca (equivalent of 1 g of HMB) in capsules, or (3) HMB-Ca (equivalent of 1 g of HMB) dissolved in water. The washout period between visits was 5–7 days. Due to the nature of the study, in which treatments of different appearances were administered, we were unable to blind the participants or the researchers involved with data collection. However, all samples were coded before being sent for analysis to control the risk of bias during sample analysis. Because we only measured outcomes that are not subjected to placebo effects, the lack of blinding is unlikely to represent risk of bias. The order of treatments was counterbalanced to control for carryover and order effects using a three-by-six (treatment-by-participant) Latin square table (www.statpages.info/latinsq.html). To randomly allocate participants to treatment sequences, participants were recruited in blocks of six, each containing three males and three females so that counterbalancing were equally in place for both sexes. The allocation sequence within each block was defined using a random sequence generator (www.random.org). All participants were fully informed about the risks and procedures involved with participation and signed the consent form before taking part in the study. The study was approved by the Institutional Ethics Committee for Human Research and complied with the declaration of Helsinki. The study was retrospectively registered at ClinicalTrials.gov (NCT05767112).

### Participants

**Fig. 1:**
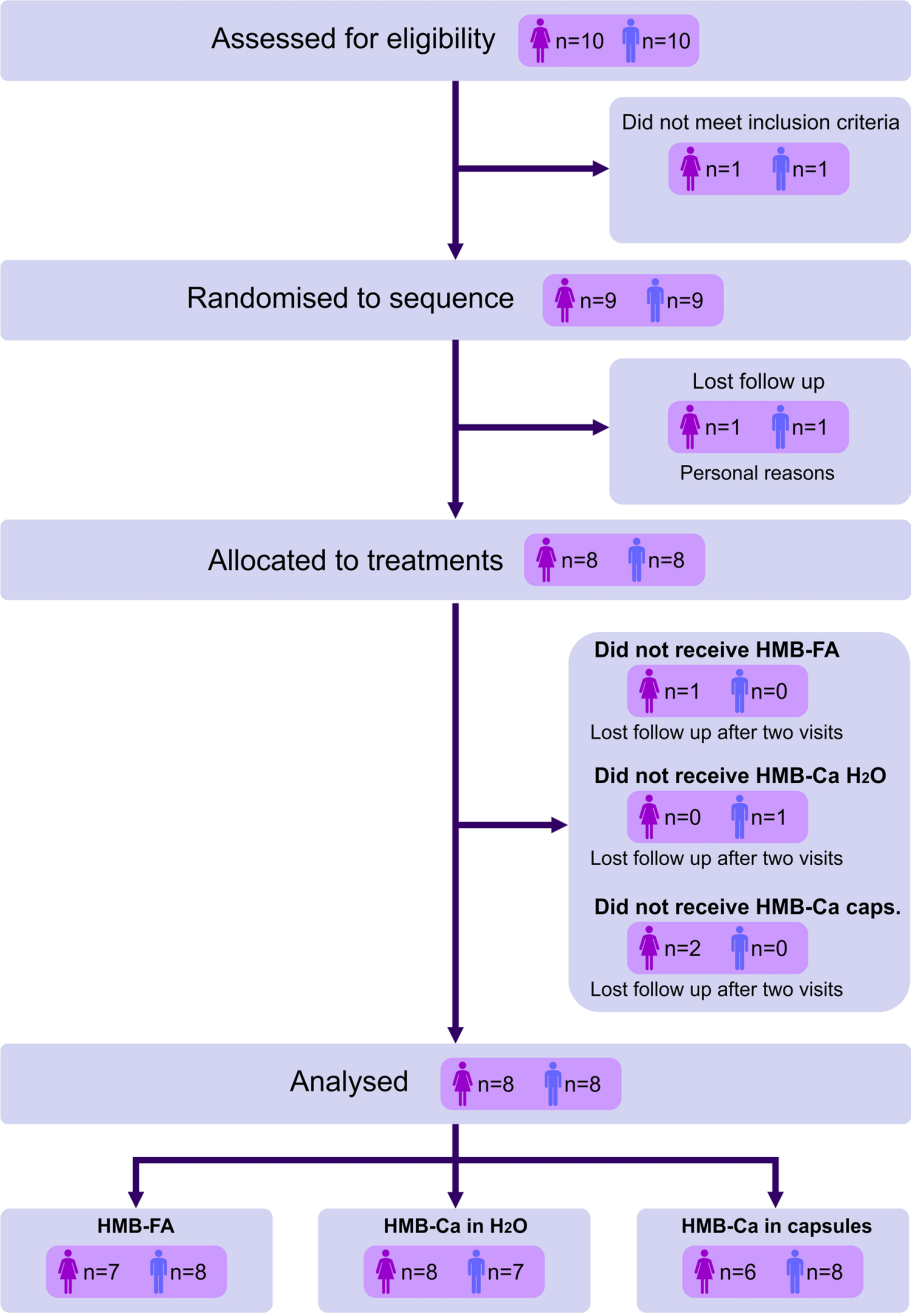
Participant recruitment flow diagram

Twenty young healthy participants were screened for eligibility, 2 of whom were not eligible. Inclusion criteria were: (1) age between 18 and 35 years, (2) ≥ 150 min of moderate to intense physical activity per week. Exclusion criteria were: (1) smoking, (2) use of statins, anti-inflammatory or any other medications that could affect lipid metabolism or blood parameters, (3) current or past use of androgenic anabolic steroids, (4) diagnosis of gastric or intestinal disorders that could affect nutrient absorption, (5) diagnosis of kidney or liver disorders that could affect nutrient metabolisation and elimination, (6) any other condition that could be perceived as a potential confounding factor or that could prevent participation in the study. Eighteen participants (females: *n* = 9; males: *n* = 9) enrolled in the study, but 2 participants (1 female and 1 male) dropped out before their first visit. Sixteen participants (females: *n* = 8; males: *n* = 8; age = 28 ± 4 years, height = 1.68 ± 0.1 m, body mass = 67.3 ± 16.6 kg, BMI = 23.6 ± 3.3 kg·m^2^) completed the study, but 4 participants (3 females and 1 male) could not visit the laboratory on one occasion (1 participant missing HMB-FA and HMB-Ca in water, and 2 participants missing HMB-Ca in capsules). Figure 1 depicts the participant recruitment flow diagram.

### Treatments and sample collection

Participants arrived at the laboratory ~ 7 a.m. following an 8–10 h overnight fast. They were instructed to refrain from alcohol and strenuous exercise in the 24 h prior to test days, and caffeine in the 16 h prior to the test days. They were also instructed to repeat the same pattern of food ingestion on all days preceding test days. A reminder to comply with all instructions was sent to their personal telephones on the day prior to all scheduled visits. Compliance with these requests was verbally confirmed on each trial.

Upon arrival, a midstream urine sample (~ 100 ml) was collected and stored at − 80° C for later analysis. A flexible catheter was then inserted in a forearm vein and kept patent using sterile saline solution. A fasting baseline blood sample was collected and then a standardised breakfast was given to all participants (energy = 418 kcal; fat = 15.5 g; carbohydrates = 60 g; protein = 10 g). Sixty minutes following breakfast ingestion, the participants took one of the following treatments: 1 g of HMB-FA in clear capsules (Muscle Tech Clear Muscle^®^, USA) provided in two clear capsules, or 1.24 g of HMB-Ca monohydrate (Millenium Sport Technologies Inc., USA) providing the equivalent of 1 g of HMB, either within two gelatine capsules or dissolved in water. All supplements were ingested with 300 ml of water. HMB-FA was ingested in clear capsules as provided by the manufacturer; HMB-Ca was encapsulated in gelatine capsules as this is suitable for powdered substances and swiftly opens in the stomach, or simply added to 300 ml of water. After the completion of the study, a sample of the HMB-FA and HMB-Ca supplements used were analysed to confirm HMB content; the analysis showed 78% of HMB content in the HMB-FA clear capsules and 95% HMB content in the HMB-Ca powder. Upon weighing the liquid contained in the clear HMB-FA capsules, we measured 0.8 g of liquid per capsule (containing 78% HMB), which allowed us to estimate that the participants in fact received ~ 1.25 g of HMB-FA and ~ 0.95 g of HMB-Ca.

Following treatment ingestion, serial blood samples were collected at the following time points: 15, 30, 45, 60, 90, 120, 180, 240, 360 and 720 min after ingestion. Since the experimental trials involved a long period of serial blood collections, we controlled food and fluid intake only in the initial 6 h following ingestion. A standardised snack (energy = 258 kcal; fat = 10.6 g; carbohydrates = 34 g; protein = 6.5 g) was given 4 h after breakfast. Two hours later, participants received a standardised main meal (energy = 700 kcal; fat = 14.5 g; carbohydrates = 76.3 g; protein = 64.8 g). Five cups of 300 ml of water were provided every 1–2 h, totaling 1.5 L up until 6 h following treatment ingestion. After the main meal (i.e. following the collection of blood sample 360 min), the catheter was removed and the participants were allowed to leave the laboratory and keep their normal activities, but returning for the last sample collection, 6 h later. They were requested not to exercise or drink any alcoholic beverage; water could be consumed ad libitum and they were instructed to eat if they wanted to, but to refrain from large meals until the collection of the last blood sample.

After treatment ingestion, participants were provided with large urine sample containers and were instructed to collect total urine for the following 24 h, returning them to the laboratory on the next day. They were instructed to keep the container refrigerated whenever possible. Upon collection, total urine volume was measured using a glass measuring cylinder, mixed thoroughly and ~ 50 ml was stored at − 80° C for later analysis. However, due to a malfunction in the freezer where some of the urine samples were stored, HMB concentrations could not be determined in 57 out of the 96 samples. The number of samples analysed in each group is as follows: HMB-FA in capsules, baseline: *n* = 7, 24 h urine: *n* = 6; HMB-FA in water, baseline: *n* = 5, 24 h urine: *n* = 8; HMB-FA, baseline: *n* = 7, 24 h urine: *n* = 6.

Blood samples of ~ 4 ml were collected using a cannula and a syringe and immediately placed on a BD Vacutainer^®^ tube containing K2EDTA, which were kept on ice for 5–10 min and centrifuged (2000*g*, 5 min at 4 °C) for plasma separation. Plasma was then placed in microtubes and stored at − 80 °C for later analysis.

### HMB quantification in plasma and urine

HMB concentration was determined in urine and plasma using stable isotope as internal standard (HMB-*d*_*8*_, Toronto Research Chemicals, Canada). Briefly, 100 μL of human urine or serum was crushed with 400 μL of acetonitrile, vortexed for 1 min and centrifuged for 10 min at 10,000*g*. Then, the supernatants were diluted and transferred to vials for LC/MS analysis. Separation was performed on an Agilent 1260 Infinity LC system consisting of an autosampler, a binary pump and a column oven. The analytical column was a Sequant Zic HILIC (100 mm × 3 mm ID, 5 μm, 200 Ao) protected with a precolumn of the same material and maintained at 25 °C. Mobile phase A was water with 5 mM ammonium acetate and mobile phase B was ACN. The analytical flow rate was set at 0.2 mL min^− 1^ and the gradient was: 97% B and hold for 1 min; ramp up to 45% over 4 min; hold for 2.30 min; bring to 97%; hold for 3.5 min.

The LC system was coupled to an Agilent 6460 triple quadrupole mass spectrometer equipped with an electrospray ionisation (ESI) source (Streaming jet), operated in negative mode and acquiring data in multiple reaction monitoring (MRM). Six level calibration curves were constructed by spiking acetonitrile or blank serum/urine samples with known HMB concentrations (3.5, 7, 14, 27, 55 and 111 µmol L^−1^). Three quality control samples (3.5, 14 and 55 µmol L^−1^) were used. The method was validated for matrix effect, accuracy and precision, and extraction recovery; it was showed to be linear in the 3–111 µmol L^−1^ range used (*R*^2^ > 0.99). Precision for intra- and inter-day assays were below 6% (RSD) for plasma and urine. Peak integration was performed using the Agilent MassHunter Quantitative analysis software and the final concentrations were corrected for the dilution factor.

### Calculation of pharmacokinetic parameters

Plasma HMB concentration curves over time were individually smoothed using the spline smoothing function to interpolate concentrations at every minute. Area under the curve (AUC) was calculated using the trapezoidal method. Peak plasma concentration (Cmax) and time to peak (Tmax) were determined by checking the smoothed curves. Half-life time (*t*_1/2_) was calculated by fitting an exponential curve (Eq. 1) to the distribution–elimination phase (i.e. from Cmax to the last data point) to find the constant of elimination (*k*) and then dividing the constant 0.693 by *k* (Eq. 2):

1 $$y=a{e}^{kx},$$

2 $${t}_{1/2}=\frac{0.693}{k}.$$

To gauge information on the rate of HMB absorption, the data relating to the absorption phase (i.e. from baseline to Cmax) was used to fit a straight-line equation, with the slope being used to indicate the rate of absorption. For the relative bioavailability calculation, HMB-Ca was set as the reference treatment as thus arbitrarily set as 100%; AUC obtained in the HMB-Ca in capsules and in the HMB-FA treatments were divided by the AUC of the reference treatment. The percentage of the total HMB dose lost in urine was calculated by multiplying the HMB concentration in urine by the total urine volume produced in 24 h.

### Statistical analysis

Plasma HMB concentrations were compared between treatments across time with a 2-factor mixed models analysis (proc mixed, SAS Studio). Fixed factors were treatment (3 levels) and time (11 levels), and participants were the random factor. Four covariance matrixes (unstructured, autoregressive lag 1, Toeplitz and compound symmetric) were tested and the best fit was determined using the Bayesian information criterion (lowest BIC value). Variance estimates and the denominator degrees of freedom were determined using the Kenward–Roger method to account for the missing data and unbalanced dataset. All pairwise comparisons were adjusted with the Tukey–Kramer correction for multiple comparisons. Plasma HMB concentration data is presented as mean values and 95% confidence intervals for sample mean (95% CI). The participants who missed trials were included in the mixed model analysis, as this is robust to deal with missing data without the need of data imputation and makes full use of the data gathered (Rosenkranz 2015).

AUC, relative bioavailability, Cmax and Tmax were compared between treatments with repeated measures one-way ANOVA with post hoc tests adjusted for multiple comparisons using the Bonferroni correction (JASP v.0.15.0.0). Only those participants who completed the three trials were included in these analyses. HMB in urine was not statistically compared within or between groups due to the low number or complete data sets. Significance level was defined a priori at 5%. Data is presented as mean ± standard deviation, unless otherwise stated.

## Results

### Plasma HMB responses to HMB-Ca and HMB-FA

**Fig. 2:**
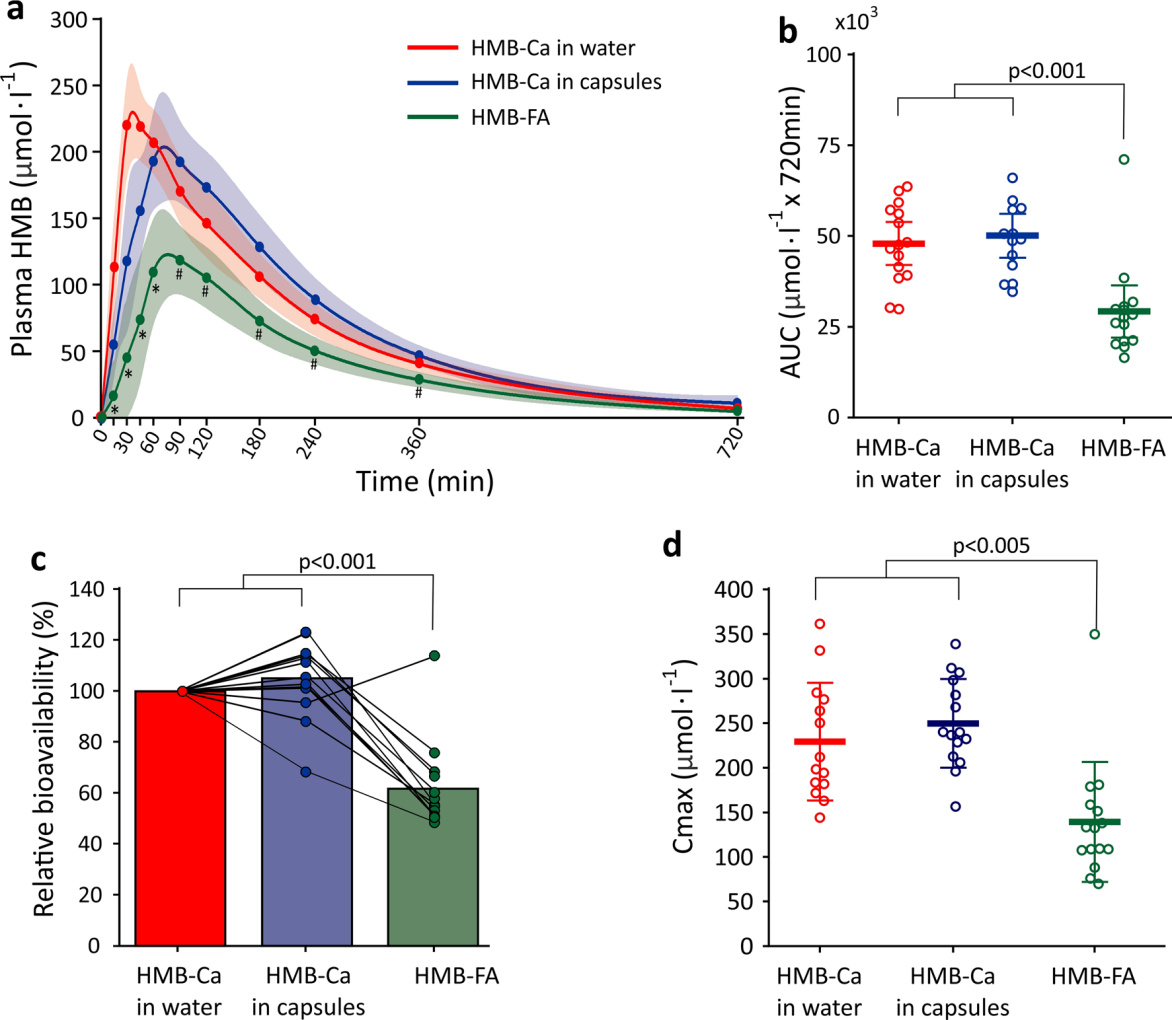
Panel a: mean and 95% CI plasma HMB concentrations in response to the equivalent of 1 g of HMB ingested in the forms of free acid (HMB-FA) or calcium salt (HMB-Ca) in gelatine capsules or dissolved in water. Dots indicate the measured values, and the solid lines represent the smoothed spline interpolation. Coloured shadows represent 95% confidence intervals of the mean. Panel **b**: area under the plasma HMB curve (AUC) for the three different forms of HMB. Panel **c**: Relative bioavailability of the different forms of HMB using HMB-Ca dissolved in water as reference and thereby arbitrarily set as 100%. Panel **d**: Peak HMB concentration in plasma (Cmax) in response to the treatments. *HMB-FA different from HMB-Ca in water (all *p* < 0.05). #HMB-FA different from HMB-Ca in capsules (all *p* < 0.05)

A rapid and large increase in plasma HMB concentrations was observed after the ingestion of all pharmaceutical forms (main effect of time: *F* = 61.34, *p* < 0.001; Fig. 2, panel A). Plasma HMB was significantly higher than baseline from the 15th min and remained elevated throughout the entire experimental period, irrespective of treatment (all *p* < 0.001; single effects of time). However, the magnitude of increase in plasma HMB was higher in both HMB-Ca forms compared with the HMB-FA form (group-by-time interaction: *F* = 2.9; *p* = 0.001). While HMB-Ca in capsules and in water showed similar values across time (*t* = − 1.24, *p* = 0.438; single effect of group), HMB-FA yielded significantly lower values vs*.* HMB-Ca in capsules (*t* = 4.86, *p* < 0.001; single effect of group) and vs*.* HMB-Ca in water (*t* = 6.21, *p* < 0.001; single effect of group). Pairwise comparisons revealed that HMB-Ca in water resulted in higher plasma HMB concentrations vs. HMB-FA at the initial 60 min after ingestion (all *p* < 0.05) whereas HMB-Ca in capsules resulted in higher plasma HMB concentrations vs*.* HMB-FA somewhat later during the 90–360 min period after ingestion (all *p* < 0.05).

Analysis of AUC revealed significantly higher exposure to HMB when ingested in the form of HMB-Ca compared with HMB-FA (*F* = 26.32, *p* < 0.001; HMB-FA vs*.* HMB-Ca in water: *t* = 6.15, *p* < 0.001; HMB-FA vs. HMB-Ca in capsules: *t* = 6.41, *p* < 0.001), but similar exposure when HMB-Ca in ingested in water or in capsules (*t* = 0.27, *p* = 0.99) (Fig. 2, panel B).

**Fig. 3:**
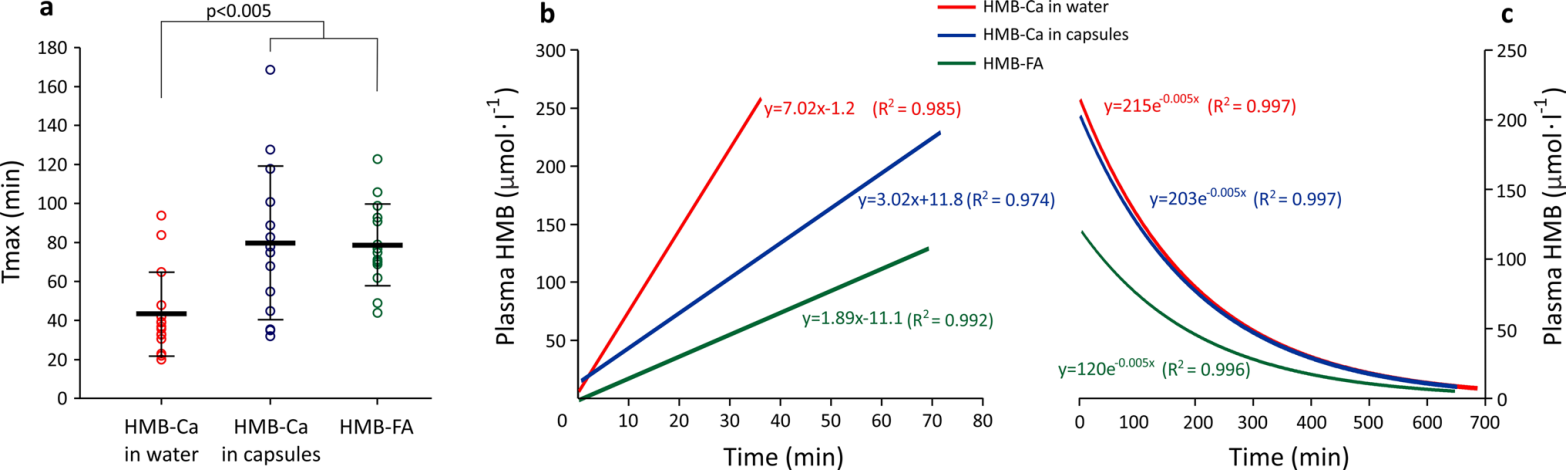
Panel **a**: Time to peak (Tmax) HMB concentrations in plasma. Panel **b**: linear regression fit for the absorption phase (i.e. from baseline to Cmax) and their respective equations and coefficients of determination. The slopes in the straight-line equations inform on the rate of HMB absorption. Panel c: exponential regression fit curves for the distribution/elimination phases (i.e. from Cmax to the last time point) with their respective equations and coefficients of determination

Relative bioavailability of HMB-FA was significantly lower than HMB-Ca (*F* = 35.35, *p* < 0.001; HMB-FA vs*.* HMB-Ca in water: *t* = 6.96, *p* < 0.001; HMB-FA vs*.* HMB-Ca in capsules: *t* = 7.57, *p* < 0.001), but no differences were shown between HMB-Ca ingested in water or in capsules (*t* = − 0.61, *p* = 1.0) (Fig. 3, panel C). Cmax was significantly higher following HMB-Ca ingestion compared to HMB-FA (*F* = 17.87, *p* < 0.001; HMB-Ca in water vs. HMB-FA: *t* = 5.80, *p* < 0.001; HMB-Ca in capsules vs. HMB-FA: *t* = 4.16, *p* = 0.001), with no differences shown between HMB-Ca in water *vs.* capsules (*t* = − 1.64, *p* = 0.347) (Fig. 2, panel D).

### Kinetics of absorption and elimination

HMB-Ca in water resulted in the fastest Tmax (*F* = 5.32, *p* = 0.013; HMB-Ca in water *vs.* HMB-Ca in capsules: *t* = 2.78, *p* = 0.03; HMB-Ca in water vs*.* HMB-FA: *t* = − 2.86, *p* = 0.027). No significant differences in Tmax between HMB-Ca in capsules and HMB-FA were shown (*t* = − 0.08, *p* = 0.99) (Fig. 3, panel A). However, examination of the slopes during the absorption phase (i.e. when plasma HMB concentrations are increasing) reveals that HMB-Ca in water led to the fastest rate of increase in plasma HMB (7.02 µmol min^−1^), followed by HMB-Ca in capsules (3.02 µmol min^−1^); HMB-FA resulted in the slowest rate of absorption (1.89 µmol min^−1^) (Fig. 3, panel B). Examination of the distribution/elimination phase and their fit exponential curves indicates virtually identical rates of elimination for all treatments. Thus, *t*_1/2_ was equally similar between treatments, being calculated as 139 min irrespective of the pharmaceutical form of HMB administered (Fig. 3, panel C).

### Urine analysis and whole body HMB retention

**Fig. 4:**
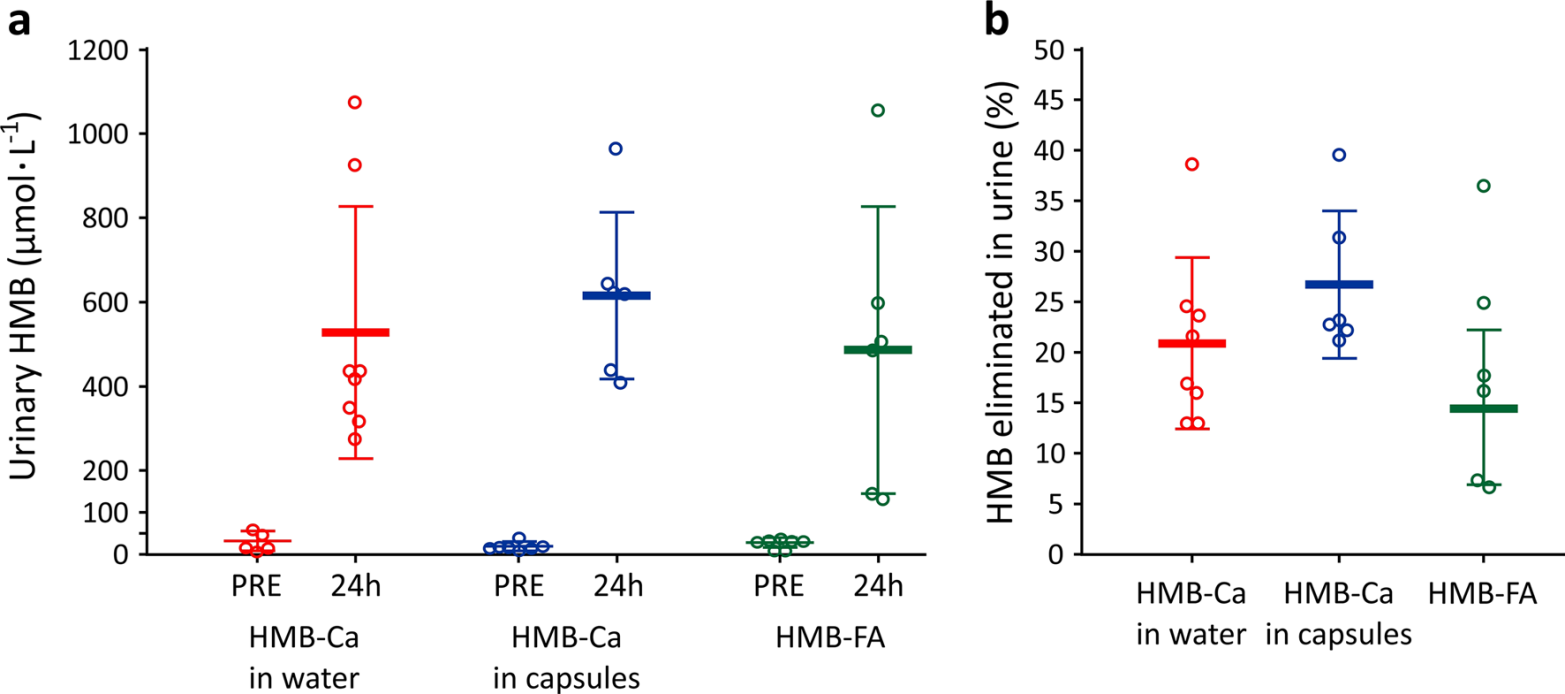
Panel **a**: Urinary HMB concentrations in midstream urine samples before HMB ingestion (PRE) and in 24 h total urine samples. Panel **b**: Percentage of the ingested dose eliminated in urine

Urine HMB concentrations increased in response to HMB ingestion in all groups. Approximately, 15–25% of the total HMB ingested was lost in urine (Fig. 4, panel B).

## Discussion

HMB has been shown to have anabolic and anti-catabolic effects in human skeletal muscle (Wilkinson et al. 2013, 2018). However, controversy still exists as to whether these properties can be translated into benefits for athletic populations, although emerging evidence suggest benefits for clinical populations under muscle wasting conditions (Tritto et al. 2019; Jakubowski et al. 2020; Phillips et al. 2022; Wu et al. 2015; Bear et al. 2019). It has been argued that the effectiveness of HMB is linked to its pharmacokinetics properties, with current knowledge indicating that HMB-FA is the preferred form due to its superior bioavailability (Wilkinson et al. 2013; Wilson et al. 2013a, b, 2014). While studies comparing the bioavailability of different forms of HMB have produced conflicting results, (Fuller et al. 2011, 2015; Shreeram et al. 2014), we sought to independently investigate whether the pharmaceutical form of HMB has any impact on its bioavailability and pharmacokinetic profile. Our data challenges the currently accepted notion that HMB-FA is superior to HMB-Ca. On the contrary, our study showed higher plasma HMB concentrations, higher AUC and higher relative bioavailability of HMB-Ca in comparison with HMB-FA. These results are aligned with previous investigations showing that HMB-Ca is highly bioavailable (Wilkinson et al. 2018).

**Fig. 5:**
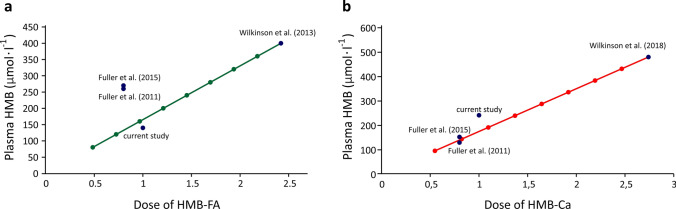
Comparison of dose–response data for HMB-FA (panel **a**) and HMB-Ca (panel **b**) using data from this study and the existing literature. The studies by Wilkinson et al. (2013, 2018) were used as reference and their data with higher doses of HMB were extrapolated to lower doses in 10% decrements. Data of the current study and that of Fuller et al. (2011, 2015) were plotted against the extrapolation-based prediction

In our study, plasma HMB concentrations following the ingestion of HMB-Ca are well within the range that could be predicted based on dose–response information available in the literature (Wilkinson et al. 2018; Fuller et al. 2011, 2015). Likewise, our HMB concentrations after HMB-FA align well with what could be predicted from data by Wilkinson et al. (2013), but the data by Fuller et al. (2011; 2015) differ from this (Fig. 5). The kinetic profile showed in our results is also comparable to previous reports, although they differ largely in terms of which form is more rapidly absorbed. We showed that both HMB-Ca and HMB-FA have similar Tmax, taking ~ 80 min to reach peak concentrations. When dissolved in water, however, HMB-Ca displays a much faster absorption profile, taking ~ 45 min to reach peak. This might suggest that the ionisation state of HMB when reaching absorption sites might affect its affinity to the transporters in the gastrointestinal (GI) tract, or simply that the time to open the capsule within the stomach and then dissolve HMB-Ca into its ions is enough to delay its absorption. Although the similar Tmax displayed between HMB-Ca (in capsules) and HMB-FA could indicate that both forms were equally available to the transporters in the GI, it is worth noting that HMB-FA took a similar time to reach a far smaller peak concentration. When analysing the slope of the line fitting the initial absorption phase, the differences in the absorption rates between treatments become clear, with faster rates for the HMB-Ca dissolved in water, followed by HMB-Ca ingested in capsules. HMB-FA, therefore, displays the slowest absorption rates. The absorption of HMB-FA is not only slower than HMB-Ca, but also less complete, which is evidenced by the lower plasma HMB concentrations and by the lower bioavailability. This suggests that, in addition to slower transport across the intestine, part of the HMB-FA is lost in the GI tract before being absorbed into the bloodstream. It is unclear, however, whether HMB is lost in its intact form in faeces due to insufficient transport across intestinal cells, or whether HMB-FA is more subjected to transformation/metabolisation within the GI tract.

To our knowledge, the mechanisms of HMB absorption through the GI tract remain poorly documented, and thus it is challenging to provide a full explanation to our results and to reconcile them with the previous literature. However, a study using an in vitro model (Ogura et al. 2019) showed that HMB can be transported into different cell types via at least three monocarboxylate transporters, namely MCT1, MCT4 and SMCT. Importantly, studies with humans show that MCT1 and MCT4 are expressed in human colon (Iwanaga et al. 2006; Gill et al. 2005) whilst SMCTs are expressed in human jejunum (Irving et al. 2016). Importantly, the study by Irving et al. (2016) showed high expression of SMCTs in human jejunum, to an extent that is similar to glucose transporters, such as GLUT2, whereas MCTs were reported to be poorly expressed in the human jejunum (Gill et al. 2005). Hence, we speculate that HMB absorption in human intestine is undertaken in the small intestine primarily by SMCTs. MCTs might also participate in HMB absorption in the large intestine where they are more abundantly expressed (Gill et al. 2005). Since SMCTs operate at neutral pH and carry out Na^+^-coupled transport in a membrane potential-dependent fashion (Ogura et al. 2019), we can speculate that the differences in absorption between HMB-Ca and HMB-FA herein shown might be due to, at least in part, their abilities to induce small changes in the pH in the small intestine. HMB-FA is a weak acid (pKa 4.4) (Ogura et al. 2019), although the HMB-FA clear capsules contain choline chloride as a buffer; HMB-Ca on the other hand is slightly alkaline. Assuming that the more acidic environment created by HMB-FA could inhibit HMB uptake via SMCTs in the small intestine, HMB would then be taken up by MCTs further in the large intestine, which could explain the delayed absorption shown after HMB-FA ingestion. This could also explain why a lower amount of HMB was absorbed following the ingestion of HMB-FA. This would be in line with the widely accepted notion that the small intestine in the preferential site of nutrient absorption (Duca et al. 2021). We nonetheless emphasise that these mechanisms are speculative and merits further investigation.

Once in the bloodstream, HMB is distributed to the peripheral tissues and eliminated by the kidneys. The elimination of HMB follows a first-order kinetics; our results showed very similar elimination kinetics for all three treatments, as evidenced by identical coefficient of elimination *k* and half-life time. These parameters are comparable to previous reports in the literature (Fuller et al. 2011, 2015). The urinary losses in our study varied between 15 and 25% of the ingested dose, which is also in alignment with the existing literature (Fuller et al. 2011, 2015). Apparently similar urinary excretion was observed between treatments. However, an important caveat is that, in addition to incomplete urinary data set, the urine HMB values were not normalised by creatinine, thereby making the HMB values herein presented susceptible to variations in hydration levels and urinary volume. We thereby emphasise that caution must be exercised when interpreting the urinary data of our study.

Taken together, our data suggest that the differences observed between HMB-Ca and HMB-FA are due to differences in the pattern of HMB absorption in the GI tract and that, once in the bloodstream, HMB is handled in a very similar manner, irrespective of the pharmaceutical form. Future studies should investigate how differences in plasma HMB and pharmacokinetic properties affect the distribution of HMB to the muscle tissue as well as the anabolic effects of HMB.

This study is not without limitations, the most important being the incomplete analysis of the urine sample set due to the loss of a substantial number of samples along with the lack of normalisation of HMB values for creatine, which hampered conclusions related to the percentage HMB dose retained in the body. Due to the nature of the experimental setup, it was not possible to employ a double-blind design; we have, however, coded all samples before sending them to analysis to ensure a bias-free quantification of HMB. The lack of measurements subjected to placebo effects also minimise the risk of bias of the open label design.

To conclude, HMB-Ca exhibited superior bioavailability and faster intestinal absorption compared to HMB-FA, whereas elimination kinetics was similar between all forms, suggesting that the pharmaceutical form of HMB affects its absorption, but not its distribution to tissues or elimination by the kidneys. Future studies are needed to confirm whether HMB calcium results in higher skeletal muscle HMB concentrations and in superior anabolic responses compared with HMB free acid.

## Data Availability

The anonymised data collected can be made available upon request.

## References

[CR1] Baptista IL, Silva WJ, Artioli GG, Guilherme JP, Leal ML, Aoki MS, Moriscot AS (2013) Leucine and HMB differentially modulate proteasome system in skeletal muscle under different sarcopenic conditions. PLoS ONE 8(10):e76752. 10.1371/journal.pone.0076752 24124592 10.1371/journal.pone.0076752 PMC3790739

[CR2] Bear DE, Langan A, Dimidi E, Wandrag L, Harridge SDR, Hart N, Whelan K (2019) β-Hydroxy-β-methylbutyrate and its impact on skeletal muscle mass and physical function in clinical practice: a systematic review and meta-analysis. Am J Clin Nutr 109(4):1119–1132. 10.1093/ajcn/nqy373 30982854 10.1093/ajcn/nqy373

[CR3] Clark RH, Feleke G, Din M, Yasmin T, Singh G, Khan FA, Rathmacher JA (2000) Nutritional treatment for acquired immunodeficiency virus-associated wasting using beta-hydroxy beta-methylbutyrate, glutamine, and arginine: a randomized, double-blind, placebo-controlled study. JPEN J Parenter Enteral Nutr 24(3):133–139. 10.1177/0148607100024003133 10850936 10.1177/0148607100024003133

[CR4] Deutz NE, Pereira SL, Hays NP, Oliver JS, Edens NK, Evans CM, Wolfe RR (2013) Effect of β-hydroxy-β-methylbutyrate (HMB) on lean body mass during 10 days of bed rest in older adults. Clin Nutr 32(5):704–712. 10.1016/j.clnu.2013.02.011 23514626 10.1016/j.clnu.2013.02.011

[CR5] Duca FA, Waise TMZ, Peppler WT, Lam TKT (2021) The metabolic impact of small intestinal nutrient sensing. Nat Commun 12(1):903. 10.1038/s41467-021-21235-y 33568676 10.1038/s41467-021-21235-y PMC7876101

[CR6] Fuller JC, Sharp RL, Angus HF, Baier SM, Rathmacher JA (2011) Free acid gel form of β-hydroxy-β-methylbutyrate (HMB) improves HMB clearance from plasma in human subjects compared with the calcium HMB salt. Br J Nutr 105(3):367–372. 10.1017/S0007114510003582 21134325 10.1017/S0007114510003582

[CR7] Fuller JC, Sharp RL, Angus HF, Khoo PY, Rathmacher JA (2015) Comparison of availability and plasma clearance rates of β-hydroxy-β-methylbutyrate delivery in the free acid and calcium salt forms. Br J Nutr 114(9):1403–1409. 10.1017/S0007114515003050 26373270 10.1017/S0007114515003050

[CR8] Gill RK, Saksena S, Alrefai WA, Sarwar Z, Goldstein JL, Carroll RE, Dudeja PK (2005) Expression and membrane localization of MCT isoforms along the length of the human intestine. Am J Physiol Cell Physiol 289(4):C846-852. 10.1152/ajpcell.00112.2005 15901598 10.1152/ajpcell.00112.2005

[CR9] Holeček M (2017) Beta-hydroxy-beta-methylbutyrate supplementation and skeletal muscle in healthy and muscle-wasting conditions. J Cachexia Sarcopenia Muscle 8(4):529–541. 10.1002/jcsm.12208 28493406 10.1002/jcsm.12208 PMC5566641

[CR10] Irving BA, Wood GC, Bennotti PN, Babu E, Deshpande A, Lent MR, Rolston DD (2016) Nutrient transporter expression in the jejunum in relation to body mass index in patients undergoing bariatric surgery. Nutrients. 10.3390/nu8110683 27801863 10.3390/nu8110683 PMC5133071

[CR11] Iwanaga T, Takebe K, Kato I, Karaki S, Kuwahara A (2006) Cellular expression of monocarboxylate transporters (MCT) in the digestive tract of the mouse, rat, and humans, with special reference to slc5a8. Biomed Res 27(5):243–254. 10.2220/biomedres.27.243 17099289 10.2220/biomedres.27.243

[CR12] Jakubowski JS, Nunes EA, Teixeira FJ, Vescio V, Morton RW, Banfield L, Phillips SM (2020) Supplementation with the leucine metabolite β-hydroxy-β-methylbutyrate (HMB) does not improve resistance exercise-induced changes in body composition or strength in young subjects: a systematic review and meta-analysis. Nutrients. 10.3390/nu12051523 32456217 10.3390/nu12051523 PMC7285233

[CR13] Knitter AE, Panton L, Rathmacher JA, Petersen A, Sharp R (2000) Effects of beta-hydroxy-beta-methylbutyrate on muscle damage after a prolonged run. J Appl Physiol (1985) 89(4):1340–1344. 10.1152/jappl.2000.89.4.1340 11007567 10.1152/jappl.2000.89.4.1340

[CR14] Kovarik M, Muthny T, Sispera L, Holecek M (2010) Effects of β-hydroxy-β-methylbutyrate treatment in different types of skeletal muscle of intact and septic rats. J Physiol Biochem 66(4):311–319. 10.1007/s13105-010-0037-3 20725872 10.1007/s13105-010-0037-3

[CR15] Mirza KA, Pereira SL, Voss AC, Tisdale MJ (2014) Comparison of the anticatabolic effects of leucine and Ca-β-hydroxy-β-methylbutyrate in experimental models of cancer cachexia. Nutrition 30(7–8):807–813. 10.1016/j.nut.2013.11.012 24984997 10.1016/j.nut.2013.11.012

[CR16] Ogura J, Sato T, Higuchi K, Bhutia YD, Babu E, Masuda M, Ganapathy V (2019) Transport mechanisms for the nutritional supplement β-hydroxy-β-methylbutyrate (HMB) in mammalian cells. Pharm Res 36(6):84. 10.1007/s11095-019-2626-3 30997560 10.1007/s11095-019-2626-3

[CR17] Oktaviana J, Zanker J, Vogrin S, Duque G (2019) The effect of β-hydroxy-β-methylbutyrate (HMB) on sarcopenia and functional frailty in older persons: a systematic review. J Nutr Health Aging 23(2):145–150. 10.1007/s12603-018-1153-y30697623 10.1007/s12603-018-1153-y

[CR18] Phillips SM, Lau KJ, D’Souza AC, Nunes EA (2022) An umbrella review of systematic reviews of β-hydroxy-β-methyl butyrate supplementation in ageing and clinical practice. J Cachexia Sarcopenia Muscle 13(5):2265–2275. 10.1002/jcsm.13030 35818771 10.1002/jcsm.13030 PMC9530546

[CR19] Prado CM, Orsso CE, Pereira SL, Atherton PJ, Deutz NEP (2022) Effects of β-hydroxy β-methylbutyrate (HMB) supplementation on muscle mass, function, and other outcomes in patients with cancer: a systematic review. J Cachexia Sarcopenia Muscle 13(3):1623–1641. 10.1002/jcsm.12952 35301826 10.1002/jcsm.12952 PMC9178154

[CR20] Rosenkranz GK (2015) Analysis of cross-over studies with missing data. Stat Methods Med Res 24(4):420–433. 10.1177/0962280214521349 24501227 10.1177/0962280214521349

[CR21] Sabourin PJ, Bieber LL (1983) Formation of beta-hydroxyisovalerate by an alpha-ketoisocaproate oxygenase in human liver. Metabolism 32(2):160–164. 10.1016/0026-0495(83)90223-8 6827986 10.1016/0026-0495(83)90223-8

[CR22] Shreeram S, Johns PW, Subramaniam S, Ramesh S, Vaidyanathan V, Puthan JK, Gelling RW (2014) The relative bioavailability of the calcium salt of β-hydroxy-β-methylbutyrate is greater than that of the free fatty acid form in rats. J Nutr 144(10):1549–1555. 10.3945/jn.114.196527 25143371 10.3945/jn.114.196527

[CR23] Tritto AC, Bueno S, Rodrigues RMP, Gualano B, Roschel H, Artioli GG (2019) Negligible effects of β-hydroxy-β-methylbutyrate free acid and calcium salt on strength and hypertrophic responses to resistance training: a randomized, placebo-controlled study. Int J Sport Nutr Exerc Metab 29(5):505–511. 10.1123/ijsnem.2018-0337 30859862 10.1123/ijsnem.2018-0337

[CR24] Van Koevering M, Nissen S (1992) Oxidation of leucine and alpha-ketoisocaproate to beta-hydroxy-beta-methylbutyrate in vivo. Am J Physiol 262(1 Pt 1):E27-31. 10.1152/ajpendo.1992.262.1.E27 1733247 10.1152/ajpendo.1992.262.1.E27

[CR25] Wilkinson DJ, Hossain T, Hill DS, Phillips BE, Crossland H, Williams J, Atherton PJ (2013) Effects of leucine and its metabolite β-hydroxy-β-methylbutyrate on human skeletal muscle protein metabolism. J Physiol 591(11):2911–2923. 10.1113/jphysiol.2013.253203 23551944 10.1113/jphysiol.2013.253203 PMC3690694

[CR26] Wilkinson DJ, Hossain T, Limb MC, Phillips BE, Lund J, Williams JP, Atherton PJ (2018) Impact of the calcium form of β-hydroxy-β-methylbutyrate upon human skeletal muscle protein metabolism. Clin Nutr 37(6 Pt A):2068–2075. 10.1016/j.clnu.2017.09.024 29097038 10.1016/j.clnu.2017.09.024 PMC6295980

[CR27] Wilson JM, Fitschen PJ, Campbell B, Wilson GJ, Zanchi N, Taylor L, Antonio J (2013a) International society of sports nutrition position stand: beta-hydroxy-beta-methylbutyrate (HMB). J Int Soc Sports Nutr 10(1):6. 10.1186/1550-2783-10-6 23374455 10.1186/1550-2783-10-6 PMC3568064

[CR28] Wilson JM, Lowery RP, Joy JM, Walters JA, Baier SM, Fuller JC, Rathmacher J (2013b) β-Hydroxy-β-methylbutyrate free acid reduces markers of exercise-induced muscle damage and improves recovery in resistance-trained men. Br J Nutr 110(3):538–544. 10.1017/S0007114512005387 23286834 10.1017/S0007114512005387

[CR29] Wilson JM, Lowery RP, Joy JM, Andersen JC, Wilson SM, Stout JR, Rathmacher J (2014) The effects of 12 weeks of beta-hydroxy-beta-methylbutyrate free acid supplementation on muscle mass, strength, and power in resistance-trained individuals: a randomized, double-blind, placebo-controlled study. Eur J Appl Physiol 114(6):1217–1227. 10.1007/s00421-014-2854-5 24599749 10.1007/s00421-014-2854-5 PMC4019830

[CR30] Wu H, Xia Y, Jiang J, Du H, Guo X, Liu X, Niu K (2015) Effect of beta-hydroxy-beta-methylbutyrate supplementation on muscle loss in older adults: a systematic review and meta-analysis. Arch Gerontol Geriatr 61(2):168–175. 10.1016/j.archger.2015.06.020 26169182 10.1016/j.archger.2015.06.020

